# Recommendations to improve patient‐centred care for ductal carcinoma in situ: Qualitative focus groups with women

**DOI:** 10.1111/hex.12973

**Published:** 2019-09-18

**Authors:** Bryanna B. Nyhof, Frances C. Wright, Nicole J. Look Hong, Gary Groot, Lucy Helyer, Pamela Meiers, May Lynn Quan, Nancy N. Baxter, Robin Urquhart, Rebecca Warburton, Anna R. Gagliardi

**Affiliations:** ^1^ Toronto General Hospital Research Institute University Health Network Toronto Ontario Canada; ^2^ Sunnybrook Health Sciences Centre Toronto ON Canada; ^3^ University of Saskatchewan Saskatoon SK Canada; ^4^ Dalhousie University Halifax NS Canada; ^5^ University of Calgary Calgary AB Canada; ^6^ University of Toronto Toronto ON Canada; ^7^ University of British Columbia Vancouver BC Canada

**Keywords:** communication, decision making, ductal carcinoma in situ, patient‐centred care

## Abstract

**Background:**

Patient‐centred care (PCC) improves health‐care experiences and outcomes. Women with ductal carcinoma in situ (DCIS) and clinicians have reported communication difficulties. Little prior research has studied how to improve communication and PCC for DCIS.

**Objective:**

This study explored how to achieve PCC for DCIS.

**Design:**

Canadian women treated for DCIS from five provinces participated in semi‐structured focus groups based on a 6‐domain cancer‐specific PCC framework to discuss communication about DCIS. Data were analysed using constant comparative technique.

**Setting and Participants:**

Thirty‐five women aged 30 to 86 participated in five focus groups at five hospitals.

**Results:**

Women said their clinicians used multiple approaches for fostering a healing relationship; however, most described an absence of desired information or behaviour to exchange information, respond to emotions, manage uncertainty, make decisions and enable self‐management. Most women were confused by terminology, offered little information about the risks of progression/recurrence, uninformed about treatment benefits and risks, frustrated with lack of engagement in decision making, given little information about follow‐up plans or self‐care advice, and received no acknowledgement or offer of emotional support.

**Discussion and Conclusions:**

By comparing the accounts of women with DCIS to a PCC framework, we identified limitations and inconsistencies in women's lived experience of communication about DCIS, and approaches by which clinicians can more consistently achieve PCC for DCIS. Future research should develop and evaluate informational tools to support PCC for DCIS.

## 1 BACKGROUND

Ductal carcinoma in situ (DCIS) is a non‐invasive breast condition characterized by abnormal cells confined to the breast ducts that represents up to 25% of all screen‐detected breast cancer.^1^, ^2^, ^3^, ^4^ DCIS itself is not life threatening, but is a risk factor for progression to invasive breast cancer.^5^ Currently, there is no reliable way to determine which cases will progress. Thus, standard treatment for DCIS is similar to treatment for invasive breast cancer: lumpectomy‐alone, lumpectomy and adjuvant radiation and/or hormone therapy, or mastectomy, options that may result in treatment‐associated adverse clinical and psychological outcomes.^5^, ^6^, ^7^ When DCIS is treated, the 20‐year breast cancer‐specific mortality rate is 3.3% (95% CI, 3.0% to 3.6%).^8^

The management of DCIS is complex for several reasons. Research shows that most women have not heard of DCIS prior to their own diagnosis, and since DCIS may not present with physical symptoms, the diagnosis is often shocking to women and their families.^9^ Women are further confused when told they have a non‐invasive condition with an excellent prognosis, yet surgical treatment is recommended.^10^ Research shows that women with DCIS desire more comprehensive information to better understand their condition and treatment options.^11^ Women with DCIS often have inaccurate perceptions of the risk of developing and dying from breast cancer,^12^, ^13^, ^14^ and experience similar levels of anxiety to women with invasive breast cancer.^15^ Given poor health‐care experiences and outcomes, women with DCIS may benefit from a patient‐centred approach.

Patient‐centred care (PCC) is defined as care that considers a patient's clinical needs, life circumstances, and personal values and preferences.^16^ PCC informs, educates and engages patients, so their perspectives are incorporated in care planning and treatment, and in on‐going medical, psychosocial and self‐management support.^17^ PCC has improved patient knowledge, communication with providers, satisfaction, treatment adherence and health outcomes; and reduced patient anxiety, missed work, readmission rates and mortality.^16^, ^17^, ^18^ Considerable research has conceptualized PCC and offers guidance on how to achieve it.^19^, ^20^, ^21^ Specific to cancer, a series of studies gathered input from patients and providers to generate a framework comprised of 6 domains that, when met, optimize PCC: fostering healing relationships, exchanging information, responding to emotions, managing uncertainty, making decisions and enabling self‐management.^22^, ^23^

Despite evidence of suboptimal care amongst women with DCIS,^9^, ^10^, ^11^, ^12^, ^13^, ^14^, ^15^ little research has examined contributing factors and, in particular, how to improve their experiences and outcomes. To that point, we found only three studies published from 1997 to 2016 that explored patient‐clinician communication about DCIS.^24^, ^25^, ^26^ In particular, no prior research investigated what women with DCIS view as PCC, knowledge essential to developing and implementing strategies, interventions or tools targeted to women and/or clinicians that support PCC for DCIS. The aim of the present study was to gain insight on how to improve PCC for DCIS by exploring whether and how women who underwent treatment for DCIS experienced the domains the comprise PCC, and if not experienced, their recommendations for how to improve PCC for women with DCIS.

## 2 METHODS

### 2.1 Approach

Given the paucity of prior research on PCC for DCIS, we employed a qualitative design to thoroughly explore women's experiences and suggestions related to PCC for DCIS.^27^ More specifically, a basic qualitative descriptive approach was used.^28^ Unlike other qualitative approaches that employ or generate theory, this technique elicits straightforward descriptions of lived experiences.^29^ We conducted in‐person focus groups rather than individual interviews because interaction amongst participants encourages rich, synergistic discussion about common and differing views.^30^ This study was approved by research ethics boards at the University Health Network and five Canadian hospitals at which focus groups were held. We complied with the 32‐item Consolidated Criteria for Reporting Qualitative Research (Appendix S1).^31^ Rigour was further ensured through purposive sampling, independent coding and review of data by the research team, reporting data with anonymous identifiers to demonstrate views from multiple participants and assessment of discrepant experiences and suggestions.^32^ There was no relationship with participants, who provided consent prior to focus groups.

### 2.2 Sampling and recruitment

We used purposive sampling by province and age to recruit English‐speaking adult women (18+ years) treated for DCIS within the previous 2 years to minimize recall bias. Women were excluded if they had been subsequently diagnosed with invasive breast cancer. Eligible women were identified and recruited through participating hospitals in five Canadian provinces where physician research team members were based. Recruitment strategies varied by hospital site dependent on local research ethics board requirements; most often, physicians linked us with their clinic nurse or research assistant, who identified eligible women from their practice and introduced the study to them either in‐person or by telephone. Thus, physicians on our research team were unaware of who from amongst their practice took part in focus groups. There was no prior relationship between focus group participants and those conducting focus groups. Women provided written informed consent in advance or at the outset of focus groups. From each of 5 provinces, we aimed to recruit 6 to 8 women, a common size for focus groups.^30^ Recruitment proceeded to thematic saturation, which was established through review and discussion of emerging themes amongst the research team concurrent with data collection and analysis.

### 2.3 Data Collection

Between April and August 2017, two‐hour focus groups were held in a meeting room at hospitals where participants were treated to provide a familiar, accessible environment. The semi‐structured focus group guide (Appendix S2) was informed by the McCormack et al PCC framework,^23^ chosen because it is the most comprehensive, specific to cancer and reflects ideal information and behaviour considered essential by cancer patients and clinicians for achieving PCC. A cancer‐specific PCC framework was chosen because prior research shows that women with DCIS are often told or believe they have invasive breast cancer^12^, ^13^, ^14^ and experience similar levels of anxiety to women with invasive breast cancer.^15^ A semi‐structured approach was employed because we wanted to understand whether women experienced PCC according to its multidimensional components, which domains were met or not met, and their suggestions for addressing distinct domains in order to optimize PCC. Women were asked to explain whether and how they experienced each of six PCC domains (fostering healing relationships, exchanging information, responding to emotions, managing uncertainty, making decisions and enabling self‐management). They were asked to focus on communication between themselves and the clinicians involved in their treatment. The first focus group was facilitated by ARG (PhD, female Scientist, training and experience in qualitative research) and observed by BN (MSc, female, Research Associate, training in qualitative research), the second focus group was co‐facilitated by BN and RU (PhD, female, Scientist, training and experience in qualitative research), and the following three focus groups were facilitated by BN. At each focus group, the facilitator reviewed the purpose of the study and focus group process. For each question, individual women were asked to speak, followed by group discussion. Focus groups were audio‐recorded and transcribed verbatim by a professional transcriptionist.

### 2.4 Data analysis

Unique themes were derived inductively from the data using constant comparison and Excel.^33^ Transcripts were analysed to identify and code all themes, and create a codebook of themes and exemplar quotes (level one); then, coded data were examined to expand or merge thematic codes, and refine the codebook (level two). Following the first focus group, BN, BJ (a research assistant) and ARG independently analysed the transcript, then compared and discussed findings to establish the codebook. It was shared with the research team, which included 7 breast cancer surgeons, whose feedback refined the codebook, and wording and flow of questions for remaining focus groups. Subsequent transcripts were analysed by BN and BJ with periodic review from ARG. The research team reviewed themes and exemplar quotes on two occasions, which led to further refinement and interpretation of themes. Quotes were tabulated by PCC domain and theme. For each domain, descriptions of information or behaviour that was present or lacking were summarized as recommendations for PCC for DCIS.

## 3 RESULTS

### 3.1 Participants

In total, 35 women participated in five focus groups (mean seven per group). Participants varied by age, treatment received and self‐reported family history of breast cancer (Table 1).

**Table 1:** Focus group participant characteristics

Characteristic	n (% of 31)^a^
Age	
30‐40	3 (9.6)
41‐50	9 (29.0)
51‐60	12 (38.7)
61‐70	5 (16.1)
>71	2 (5.7)
Province	
British Columbia	10 (28.6)
Alberta	5 (14.2)
Saskatoon	6 (17.1)
Ontario	8 (22.8)
Nova Scotia	6 (17.1)
Treatment received within 2 years of focus group	
Lumpectomy‐only	1 (3.2)
Lumpectomy + Radiation	16 (51.6)
Mastectomy	14 (45.1)
Family history of breast cancer	
Yes	12 (38.7)
No	19 (61.2)

a While 35 women participated, demographic data are missing for 4 women due to site‐specific research ethics requirements

### 3.2 Themes by PCC domain

Themes and exemplar quotes by PCC domain are included in Appendix S3, summarized in Table 2 and discussed here. Themes were similar across age groups, treatment received and province, suggesting that our findings reflect the experiences of women with DCIS across Canada.

**Table 2:** Themes and exemplar quotes by PCC domain^23^

PCC domain^23^	Theme	Exemplar quotes
Fostering healing relationships	Honesty	He said, I’ve not had a patient diagnosed with this before. So I don't know what I don't know yet. So I found that very, very, very helpful in that he was honest to say, like I don't really know what I don't know yet. (AB)
	Patience	She was really good for me. She took the time to explain things and I didn't feel like I was rushed even though I know her caseload is very heavy. So for me that worked well. (SK)
	Competence	There was a sense of trust that I had with her…I felt that she knew what she was doing, she was going to do it the best she could. There was a sense of trust that I had with her because of that. (NS)
	Non‐domineering body language	He sat up on the examining table, I was in the chair and he proceeded to be the expert which he is…but was talking to me like I was just going to be another small piece of his day as we went on. He would make decisions and move on. I wasn't overly impressed. (SK)
	Personal enquiries	Great bedside manner…I think she talked about her family and I’m a vet, and so we talked about that and her dog and you know kind of made it all…made it all quite light and comfortable (AB)
Exchanging information	Variable language to describe DCIS	The first time it was explained to me as the stage‐0 and he said something like if you have to get a cancer this is a really good one. (AB)
	Repeating and summarizing information	She was incredibly patient with me and giving me the information and I wasn't receiving it well at one point and she just repeated it. (ON)
	Guidance with questions	I didn't even know what questions to ask. You know you know so little. You don't even know what you don't know. (SK)
Responding to emotions	Little response from physicians	I was emotional…but my doctor's really busy so she just gave me the bad news and off I went after. (BC)
	Women reluctant to express emotions	Only now, almost a year later I’m starting to actually find that it's bothering me more than it did. At the time I just went into survival mode and it was like, I’m going to act like a normal person and pretend this isn't happening. (AB)
	Little supportive care	Her reaction was very numb. She was very uncomfortable…I was back in my car 5‐minutes later calling my husband crying, right? Like…and that's, no support from her… so that was frustrating (BC)
Managing uncertainty	Little information about prognosis	I’m not sure that he described any uncertainty with it…this is what it is, like very factual, and this is what we need to do (NS)
Making decisions	Little involvement in discussion or decisions	They don't say, hey are you a marathon runner? Are you this? Are you that? Do you go to the gym? Do you lift your kids? They don't ask you any of that (BC)
	Uninformed and frustrated	I brought up mastectomy because I wanted to know. And she just simply said, it's not appropriate for you which I was a little, honestly… but I wanted it to be appropriate for me, you know because still to this day, I don't know, I’m not really that thrilled with radiation, right? To be honest. But I heard her and we proceeded. (NS)
Enabling self‐management	Little information about follow‐up or self‐care	My experience was, you're done treatment, bye‐bye. That's it. I was not happy about that. It would have been nice if she had said to me, you are now done, you're going to feel such and such. (ON)

#### 3.2.1 Fostering healing relationships

Most women reported having a good relationship with their clinician. Women described several clinician characteristics and behaviours they viewed as fundamental to having a good relationship. Characteristics were identified by the themes of honesty, competence and patience, and behaviours were identified by the themes of non‐domineering body language and personal inquiries.

##### Honesty

Two women said their family physician acknowledged his or her gaps in knowledge about DCIS and expressed commitment to them to learn the necessary information, which reassured women about the dedication of their physician to finding accurate information to address their questions and concerns.

##### Competence

Several women said that having trust in clinician's technical competence provided them with a sense of security during a state of vulnerability, and with reassurance that they would be taken care of. One woman said that knowing her surgeon was ‘the best’ was more important than having a surgeon that was personable.

##### Patience

All women said that clinician patience during their discussion was imperative to a positive relationship. They described patience as having a calm demeanour, and providing information and answering questions in an unrushed manner. This made women feel listened to and cared for. Women who were rushed by the clinician during their appointment said they felt isolated and upset.

##### Non‐domineering body language

Several women emphasized the role of body language in communication. Women said that their clinician expressed caring and commitment by sitting beside them and at the same eye level. Alternatively, one woman said that, by standing or sitting at a higher point than her and talking physically down to her during the entire discussion, the clinician made her feel insignificant.

##### Personal inquiries

Most women valued when clinicians spent time to inquire personally about them prior to discussing clinical details. By taking time to get to know them, women said this reduced anxiety and made them feel more at ease to discuss concerns and ask for support.

#### 3.2.2 Exchanging information

Most women were confused by unclear and conflicting terminology used by clinicians to describe DCIS, which also failed to distinguish DCIS from invasive breast cancer. Several women said their understanding improved when clinicians took time to explain and repeat information, and some women desired guidance on which questions to ask.

##### Variable language to describe DCIS

Women said their clinician used multiple terms to describe DCIS including ‘early‐stage breast cancer’, ‘pre‐cancer’ and ‘abnormal cells’, terms that led them to believe they had cancer rather than a pre‐malignant condition. Several women said they were given conflicting information about their diagnosis by different clinicians, for example their surgeon and their radiation oncologist, throughout their care. Women felt that multiple terms and discrepancy in language between clinicians amplified confusion and misunderstanding of their condition.

##### Repeating and summarizing information

Several women said they appreciated that their clinician took time to repeat information and to summarize the information discussed at the end of their appointment. They said this helped them feel more comfortable with their clinician and helped to consolidate information.

##### Guidance with questions

Several women said they were unprepared to know what to ask, making it difficult to engage in discussion, and be an advocate for their needs and preferences. They suggested that information tools or education about what to ask would help them communicate with clinicians.

#### 3.2.3 Responding to emotions

Nearly all women felt emotional about their diagnosis but many withheld emotions from their physician. Those that did experience an emotional reaction said their physician did not enquire about, acknowledge or offer emotional support, and few were referred to supportive care.

##### Little response from physicians

Many women conveyed an emotional reaction to their physician. Amongst those, most said their emotions were not acknowledged. A few said their physician addressed concerns by emphasizing the slow progression to invasive cancer and good overall prognosis, information that women said was important for reducing anxiety.

##### Women reluctant to express emotions

Several women said they did not convey an emotional reaction to their physician, but provided differing reasons: they did not know if it was appropriate or acceptable to discuss those feelings, they felt uncomfortable sharing those feelings, they were overwhelmed with a DCIS diagnosis, and the emotional reaction was delayed to after the appointment, and they felt guilty for feeling emotional given that clinicians and family said ‘they were lucky to just have DCIS’ and that ‘DCIS was the best cancer to have’.

##### Little supportive care

Following physician consultation, few women were directed to meet with or were given contact information for a nurse or patient navigator to discuss their concerns. During and following treatment, several women desired psychological support. These women said that during treatment they focused on survival and next steps, while after treatment, when they had time to process what happened, they felt emotional and experienced psychological distress. Women in need of psychological support following treatment reported that physicians did not ask them about emotions during follow‐up appointments, and they were unaware of how to access DCIS‐specific resources such as a support group or psychological counselling on their own.

#### 3.2.4 Managing uncertainty

##### Little information about prognosis

Nearly all women had misconceptions about the progression of DCIS to invasive breast cancer, in part because DCIS was described using cancer‐related terms (ie pre‐cancer) and because they were given little to no information about the likelihood of progression. As a result, women thought they already had cancer or that progression was inevitable, contributing to confusion and anxiety. Two women given brief information about the risk of DCIS progressing to invasive disease said this helped them understand the purpose and high chance of successful treatment.

#### 3.2.5 Making decisions

Few women reported being engaged by their physician in treatment decision making. They felt obliged to comply with physician recommendations, uninformed about treatment benefits and risks, and frustrated with the lack of information or education to help them to participate in their care.

##### Little involvement in discussions or decisions

Few women were asked about lifestyle and preferences for recovery, or involved in discussing or choosing treatment. Instead, most women said their physician told them what treatment they would receive, or described treatment options but strongly recommended one option with little rationale or discussion about how that option matched women's preferences.

##### Uninformed and frustrated

Given no option to participate in discussions or decisions about treatment options, women said they felt obliged to comply with physician decisions or recommendations even if it was not their personal preference. Women felt uninformed about the possible benefits and risks of treatment, which left them frustrated. They emphasized the need for DCIS information and education that would better prepare them for involvement in decisions.

#### 3.2.6 Enabling self‐management

Little information about follow‐up or self‐care.

Many women described an abrupt termination from interaction or communication with clinicians following treatment, which led to feeling isolated and unsupported. Most women said they were not given information about a follow‐up plan, self‐care advice or who to contact if they experienced side‐effects or other concerns, so they felt unprepared to manage their health and confused about on‐going care. A few women were given contact information of a patient navigator or nurse, which they found helpful in addressing concerns following treatment.

### 3.3 Recommendations to achieve PCC for DCIS

Table 3 summarizes recommendations to achieve PCC for DCIS derived from women's accounts of information or behaviour that was present and appreciated, or absent and desired. Fostering a healing relationship was achieved for all women through a variety of approaches. For all other domains, few women offered examples of information or behaviour that addressed that domain. Instead, by describing information or behaviour that was lacking, they highlighted numerous approaches by which clinicians can more consistently achieve PCC for DCIS.

**Table 3:** Recommendations for clinicians to achieve PCC

PCC domain^23^	Current study	
	Present	Absent
Fostering healing relationships	Be honest about gaps in knowledge Convey or describe clinical competence Employ non‐domineering body language (ie sit at same level) Make personal inquires to establish rapport Exhibit patience (ie calm demeanour, unrushed manner)	–
Exchanging information	Take time to explain, repeat and summarize information	Use consistent terms to describe DCIS Distinguish DCIS from invasive breast cancer Ensure that different clinicians provide non‐conflicting information Provide ‘question prompt’ tools to help women prepare for discussions
Responding to emotions	Emphasize the low likelihood or slow progression to invasive cancer and good overall prognosis Refer women to, or provide contact information for supportive or psychosocial care	Encourage women to express emotions or concerns when diagnosed, and through treatment and follow‐up appointments Acknowledge and validate an emotional reaction to DCIS At the time of diagnosis and thereafter, ask women if they are interested in speaking to a nurse, patient navigator, or counsellor for information or psychological support
Managing uncertainty	Explain the purpose of treatment using information about the risk of DCIS progressing to invasive breast cancer	Provide information about the likelihood of progression to invasive breast cancer
Making decisions	Outline the benefits and risks of treatment options and invite women to choose a preferred option	Describe the benefits and risks of treatment options Ask women about lifestyle and personal preferences that could influence treatment choice Provide information or resources to help women participate in treatment discussion and decision making
Enabling self‐management	Provide contact information for patient navigator or nurse to assist with follow‐up support	Provide a clinical follow‐up plan Provide or refer women to self‐care advice Identify who women should contact about side‐effects or concerns

## 4 DISCUSSION

Women of all ages from across Canada who received various treatment for DCIS said that their clinicians used multiple approaches to achieve the PCC domain of fostering a healing relationship, but most women described an absence of desired information or behaviour to achieve the PCC domains of exchanging information, responding to emotions, managing uncertainty, making decisions and enabling self‐management. By comparing the accounts of women with DCIS to a 6‐domain cancer‐specific framework of ideal information and behaviour considered essential by cancer patients and clinicians for achieving PCC,^23^ we identified limitations and inconsistencies in women's lived experience of communication about DCIS. At the same time, women identified or suggested approaches by which clinicians can more consistently achieve PCC for DCIS. Those recommendations are consistent with national consensus recommendations on PCC for DCIS generated by Delphi process involving 30 clinicians of multiple specialties who manage DCIS and 32 DCIS survivors.^34^

The findings give rise to several implications for policy, practice and on‐going research. Women in our study were confused about the variety of terms used by their clinicians to describe DCIS and as a result thought they had breast cancer. Their confusion was compounded when different clinicians involved in their care used conflicting terms. Previous research also found that patients have difficulty understanding DCIS^9^ and that clinicians have difficulty describing DCIS.^35^ Other research found that when terms for DCIS involved the word ‘cancer’ (ie stage 0 breast cancer), which is commonplace,^24^, ^25^ compared with terms such as ‘abnormal cells’, women experience more psychological distress and prefer more aggressive treatment.^36^, ^37^, ^38^ Clearly, efforts are needed to improve patient‐clinician discussions about DCIS. One way to achieve this is to clarify the language used to describe DCIS. As was done for other pre‐cancerous tumours,^39^ further research is needed to engage international experts in revisiting cancer staging and classification and assessing if or how to change the DCIS name. Efforts are also needed to ensure consistency in language across differing specialties. In other research, we found that multidisciplinary teamwork was enhanced when coordinated through integrated programs, staff were co‐located, patient navigators mediated patient transitions between clinicians, and other activities such as team meetings supported clinician efficiency.^40^ Future research could investigate whether these or other strategies could harmonize DCIS language.

Participating women were also frustrated with the lack of information or education on DCIS. This was relevant to most PCC domains; for example, they did not know what to ask when exchanging information, who to turn to for emotional support, their prognosis based on the likelihood of DCIS progressing to invasive breast cancer, the benefits and risks of different treatment options or why a particular option was recommended to them, a post‐treatment follow‐up plan or self‐care strategies. As a result, they were unable to fully engage in their own care. It is well established that newly diagnosed cancer patients require adequate information in order to understand their diagnosis^41^, ^42^, ^43^; other research shows that the information needs of women with DCIS are largely unmet.^44^ In other research, we examined the content of 39 DCIS informational resources available to women on the Internet and found they employed confusing labels for DCIS and poorly addressed PCC domains, and few were assessed as high quality.^45^ Future research should develop and evaluate DCIS information tools that women and clinicians can use to improve communication about DCIS. As patient recall of medical information conveyed by clinicians is often inaccurate,^46^, ^47^ DCIS information tools could also supplement and reinforce clinician information.

Women with DCIS in our study and elsewhere^11^ desired more involvement in discussions and decisions. Providing time for women to ask questions is important but insufficient for those who are uncertain of what to ask.^48^ Question prompt lists are a relatively inexpensive strategy for facilitating communication between patients and providers.^49^ Lo and colleagues identified the questions most women with DCIS wanted answered,^50^ which could form the basis of a DCIS question prompt tool, prior to testing of the impact on patient‐important outcomes such as perceived PCC, knowledge of DCIS and satisfaction with the patient experience. As the vast majority of women treated for DCIS will become long‐term survivors,^8^ issues around follow‐up and quality of life are of particular importance. Consistent with previous research,^51^, ^52^, ^53^ women in this study experienced distress at the time of diagnosis, during treatment and well‐after treatment, which reduced health‐related quality of life. Participating women said few clinicians asked about, acknowledged, or responded to emotional concerns, emphasizing the need for self‐management informational resources that women can refer to in their own time. Research by Haq and colleagues^54^, ^55^ developed such a resource for women with breast cancer, which was shown to significantly improve patient self‐efficacy [44.12 vs 44.66, *P* = .046], a key measure for patient self‐management.^56^ As DCIS is distinct from invasive breast cancer, future research should develop and evaluate self‐management informational tools for women with DCIS.

This study has several strengths. It is the first wide‐scale, qualitative study to explore PCC for DCIS as a means of identifying how to improve patient‐provider communication. We employed robust qualitative research methods,^27^, ^28^, ^29^, ^30^ adhered to standards for qualitative research^31^ and used techniques to optimize rigour.^32^ We sampled women with a wide variety of characteristics to enhance the reliability and validity of the findings, and involved the research team, largely comprised of breast cancer surgeons, to further validate and interpret the findings. We also transformed the findings into tangible recommendations that can be broadly applied to improve PCC for DCIS. By employing a rigorously developed, cancer‐specific, comprehensive PCC framework,^23^ we compared the experience of participating women to what may be considered an ideal model of PCC. A few limitations may influence the interpretation and application of these findings. All participants were recruited from urban hospitals; thus, the findings may not be transferrable to women in rural or remote settings. Participants may be particularly interested in PCC, and their views may not be transferrable to other women. Participants were Canadian, so the findings may not be transferrable or relevant to women with DCIS or clinicians in other jurisdictions. A few women underwent surgery for DCIS up to two years prior to our focus group; thus, their accounts may have been influenced by recall bias.^57^ As noted earlier, due to research ethics board requirements that limited access to demographic information, we lacked certain information such as ethnicity, and the characteristics of 4 participants were unable to be matched to responses. As is typical of focus groups, exemplar quotes are identified only by the focus group location and not by characteristics of individuals; while this may limit readers’ ability to distinguish between statements, key findings were consistent amongst women. Here we did not consider clinician experiences; we concurrently interviewed clinicians about PCC for DCIS and will report those findings elsewhere. Despite these limitations, we transformed findings into tangible recommendations that could be validated and then widely applied elsewhere to improve PCC for DCIS.

## 5 CONCLUSION

Although previous DCIS research identified persistent patient‐clinician communication challenges, this study was the first to compare the experiences of women with DCIS to an established ideal of PCC. This identified information or behaviour that was present and appreciated, or absent and desired. These experiences were transformed into recommendations, offering clinicians numerous approaches to more consistently engage women and achieve PCC for DCIS by fostering a healing relationship, exchanging information, responding to emotions, managing uncertainty, making decisions and enabling self‐management. Future research is needed to continue improving patient‐clinician communication by considering a name change for DCIS, and by developing and evaluating information tools that can support PCC.

## CONFLICT OF INTEREST

None to declare.

## INFORMED CONSENT AND PATIENT DETAILS

I confirm all patient/personal identifiers have been removed or disguised so the patient/person(s) described are not identifiable and cannot be identified through the details of the story.

## Data Availability

All data are included in the manuscript and accompanying supplemental files.
