# Supplementary material for: Recommendations to improve patient‐centred care for ductal carcinoma in situ: Qualitative focus groups with women
Source: Health Expect. 2019 Sep 18;23(1):106–14. doi: 10.1111/hex.12973 (PMC6978860; doi:10.1111/hex.12973)
Supplement: Supplementary file 2 [file HEX-23-106-s002.docx]

**Supplemental File 2. Focus group guide**

**INTRODUCTION**

- Thank you for participating (introductions)
- Research has found that women are confused whether DCIS is cancer or not, and why they need treatment, and that physicians find it challenging to explain DCIS to patients
- The overall aim of our study is to understand how to improve communication between patients and physicians about DCIS
- The purpose of today’s meeting is to learn what was satisfying and less satisfying about the discussions you had with your physician, and your recommendations for improving the experience for future women diagnosed with DCIS. We are interested in learning about your expectations, were they met, what was done well, and what could have been done better.
- Do you have any questions before we begin?

**FOSTERING PATIENT-PHYSICIAN RELATIONSHIP**

How did the physician who treated you establish a friendly, courteous and comfortable relationship with you?

**EXCHANGING INFORMATION**

What words or language did your physician use to explain DCIS?

**RESPONDING TO PATIENT EMOTIONS**

You may have experienced emotions or concerns upon learning you had DCIS. How did your physician gauge and respond to your reaction?

**MANAGING UNCERTAINTY**

Women with DCIS are often confused about whether DCIS is cancer or not, and why they need treatment. How did your physician explain the chances of DCIS turning into invasive breast cancer or recurring?

**MAKING DECISIONS**

Women with DCIS can undergo lumpectomy with radiation, or mastectomy, with or without breast reconstruction. How did your physician involve you in discussing and choosing a treatment option?

**ENABLING PATIENT SELF-MANAGEMENT**

How did your physician prepare you for next steps, and to manage your own health and well-being after treatment was completed?

**OVERALL**

What else would have been helpful to you during your care or afterwards? Was there anything that your physician could have done or that the health system could have done to improve your care or support you better?

**CONCLUSIONS**

Thank you for participating
