# Supplementary material for: Recommendations to improve patient‐centred care for ductal carcinoma in situ: Qualitative focus groups with women
Source: Health Expect. 2019 Sep 18;23(1):106–14. doi: 10.1111/hex.12973 (PMC6978860; doi:10.1111/hex.12973)
Supplement: Supplementary file 3 [file HEX-23-106-s003.docx]

**Supplemental File 3. Themes and exemplar quotes**

| PCC domain [23] | Theme | Quotes |
| --- | --- | --- |
| Fostering healing relationships  *Patient-clinician relationships are built through open communication, trust in clinician competence and demonstrating caring and commitment.* | Honesty | I’ve not had a patient diagnosed with this before. So I don’t know what I don’t know yet. So I found that very, very, very helpful in that he was honest to say, like I don’t really know what I don’t know yet. (AB)  she was always available and she said, I don’t know enough about this myself, so if you have any questions, I will do the research. (AB) |
|  | Perceived competence | there was a sense of trust that I had with her…I felt that she knew what she was doing, she was going to do it the best she could. There was a sense of trust that I had with her because of that. (NS)  There was a sense of connectedness between us as well; trust on both sides that I would follow directions and follow-up and attend to whatever needed to be done. (NS)  in my experience is one of comfort but I think there was that sense of yah, I’m in good hands, that’s the whole thing. I’m in good hands with this physician (NS)  I always feel comfortable if I feel like I can trust them. And they’re very you know they portray that they know what’s happening and this is gonna be okay. Or you know these are the things you need to do. (BC)  I think for me it was that she was confident. She was very articulate… And I just felt she knew what she was talking about without being condescending or arrogant. (AB) |
|  | Non-domineering body language | The initial discussion did not happen across any desk. I can recall her coming and sitting down right beside me. That physical dynamic was very personable. (ON)  she took a stool and sat down right below me…and asked ‘are you understanding any of this’? Don’t worry, we’ll look after you. (ON)  I got the diagnosis from my family doctor and she had been trying to call me for about two or three days and we were missing each other. So I happened to have my daughter into the clinic for an appointment and she saw me and she came to me in the hallway and she put her arm around me. So I’m like, okay, this is probably not gonna be good today. it gave me a few minutes to kind of think about how I was gonna handle the whole situation with my daughter there and…because if she wouldn’t have done that in the hallway, I wouldn’t have known what was gonna come and how I was gonna you know get her out of the room. (NS)  you know good eye contact, came closer to me, caring, right? (NS)  He sat up on the examining table, I was in the chair and he proceeded to be the expert which he is…but was talking to me like I was just going to be another small piece of his day as we went on. He would make decisions and move on. I wasn’t overly impressed. (SK) |
|  | Personal enquiries | Great bedside manner…I think she talked about her family and I’m a vet, and so we talked about that and her dog and you know kind of made it all…made it all quite light and comfortable (AB)  she did introduce herself and she found out what kind of work I do… she found out a lot about me (ON)  she made feel very comfortable right away and we had talked about clothes and wine…it was very comforting to feel a little bit normal at that point. (AB) |
|  | Patience | She was really good for me. She took the time to explain things and I didn’t feel like I was rushed even though I know her caseload is very heavy. So for me that worked well. (SK)  there was no feeling of you know I’ve got other people waiting. I remember one of the more challenging discussions was well over an hour in her office. And I remember feeling …wow, she’s really wanting to make sure that we feel comfortable. (ON)  I was pretty floored. So she took the time, she recognized that. she took the time to spend with me and explained (NS)  So I went in a little informed but she broke down everything else and she…we kind of went through the report together. Yah, so just her taking the time is I think where that compassion comes from. (NS)  my doctor is…you can just sit down and you could just talk with her and she always has time for you which is really difficult to find now-a-days. (BC)  …she was extremely patient with me (ON)  Dr. X is very, what I call present to me in this being that presence, a presence that you could feel and sense (NS) |
| Exchanging information  *Exchanging information encompasses reciprocal sharing of knowledge, beliefs and recognizes patient values and preferences to create a shared understanding.* | Variable language to describe DCIS | If it’s not cancer, then let’s not call it cancer. Well, carcinoma is cancer. I mean, it’s just not invasive right? I don’t understand the pre-cancer language, I don’t. (BC)  The first doctor described it as cancer, so from the time I got the first diagnosis until coming to see my surgeon I did my own researching and my surgeon described it as pre-cancer and basically not a problem, nothing to worry about. (BC)  I was told that I had DCIS… I was told it was…it was a form of cancer but it was just contained (ON)  they said it was contained. I was told that it was not cancer. (ON)  this is just something that is abnormal but benign, we’ll keep an eye on it… second time around she says, okay now we have DCIS and things are different… She talked about it as being a cancer stage-0… I remember her being very clear it is classified as carcinoma but it is this very early…then there was the next appointment with the surgeon to re-visit everything. (ON)  I was never told that it wasn’t cancer… what I remember, they just said, I was stage-0… it was like very, very early stage of cancer. (ON)  my surgeon was very good in explaining it and at no point did she way it was cancer. She said, it’s sort of a prelude to what could be but not that it was (ON)  My surgeon… told me it’s a cancer but its stage-0. It’s the first stage of cancer…but as long as it is inside the ducts it’s not considered as invasive (ON)  Well, she said, you have cancer but I don’t think its cancer; like it was sort of like fake cancer; do you know what I mean? That was sort of the way it was…(BC)  Dr. X explained it really well, like in detail. She went through it in segments and told me, it’s malignant but it’s capsulated. Which means it hasn’t spread. you know everything was explained to me the way I understood it and they allowed me to voice what my concerns are you know. (BC)  she told me when I came for one of my meetings with her and she kept on saying, it’s pre-cancer. I said it’s pre-cancerous. I did not clue into that until I got home and I was talking to my husband and I said, well she kept on saying pre-cancerous. (SK)  said it was, I had abnormal cells but it wasn’t cancer it was pre-cancer (SK)  Well it really wasn’t explained at all, I mean it just said, oh you have a lot of calcium in your breast and they showed me the x-ray, like in the, when I had the…all the calcium and all these other things that’s all the cancer cells. (SK)  what you have cancer of the breast but you’re very lucky that it’s this because we can help you a lot more than if it had been another kind of cancer (ON)  you have two spots… now they’ve grown a little bit. So its hardening and we need to get rid of them so that because they’re…we found some cancer cells, they might spread to your breast and then cause you to have cancer everywhere else. So she was very thorough in explaining but she did not use the word it’s not cancer. (ON)  you have cancer of the breast but it’s called that in situ (ON)  I came back for a check-up and they said, I had DCIS… he said that it was cancer. (NS)  So my family physician said, you know you have cancer and you need to …because of your family history and because of like the grade and everything else and multiple sites and blah, blah, blah. And Dr. X, she didn’t call it pre-cancer at all during that initial consultation before the surgery. Because I think the fear was is that like my mom, it had become invasive. So I was under the impression that I did have cancer. (BC)  the first time it was explained to me as the stage-0 and he said something like if you have to get a cancer this is a really good one. (AB)  when my family physician called me into actually give me the diagnosis, he used the word cancer. So that was also very alarming… I remember my physician saying to me, my family physician saying, it is cancer but the good news is that it doesn’t appear to have moved to your lymph nodes. (AB)  Well the first doctor, described it as cancer and he had no knowledge really. So from the time that I got the first diagnosis til coming to see Dr.X, I did my own researching. And then Dr. X described it as pre-cancer and basically not a problem but nothing to worry about. So that changed my perception of it to some degree. (BC)  I had been told that it’s not cancer and …I’ve been told that at the same time it is a stage-0. (ON)  the doctor that did the radiation …said, well but it’s not cancer. So… I was kind of confused at that time because my surgeon never told me that it wasn’t cancer. The information was somebody else was saying something and somebody else was saying something else. (ON)  I sat with the physician, he did the biopsy and he said to me was you’re gonna need surgery but he didn’t use any terminology to describe exactly what it was for. So I was a little bit frightened by that. Then when my family physician called me into actually give me the diagnosis, he used the word cancer. So that was also very alarming. It wasn’t until I met my surgeon that the term DCIS was actually discussed. So up until that point, I thought I had cancer, cancer. And I remember my physician saying to me, my family physician saying, it is cancer but the good news is that it doesn’t appear to have moved to your lymph nodes. So I was trying to grab…context with that because I just had no idea what was going on. So when I talked to Dr. X she explained you know the stage-1 but then there was the grading system as well which also confused me because I was told I was a grade-3. So I was really kind of thinking that this is really bad but really good. So it was really confusing to be honest. But after speaking with Dr. X a little bit and with her…my nurse navigator, I started to kind of understand what was happening. But there was a period of time there like I literally had no idea what was going on and I remember talking to my mom and was thinking I’m getting like a mastectomy and it went to one extreme then back to the other. So it seemed like it was gonna be really, really, really, really serious and then all of a sudden it seemed like okay, well maybe this is a manageable thing. But that period of time was probably one of the most stressful periods of my life because I just didn’t know what was going on. So it was pretty scary. (AB) |
|  | Repeating and summarizing information | she was incredibly patient with me and giving me the information and I wasn’t receiving it well at one point and she just repeated it. (ON) |
|  | Guidance with questions | I didn’t even know what questions to ask. You know you know so little. You don’t even know what you don’t know. (SK)  Sometimes it’s hard to be an advocate when you don’t know the questions to ask. (BC) |
| Responding to emotions  *Helping to identify emotions, while assessing, validating, expressing empathy, and providing support.* | Little response from physicians | I was emotional…but my doctor’s really busy so she just gave me the bad news and off I went after. (BC)  just basically read my results and told me I the unlucky one and had cancer which wasn’t really the right answer (BC)  they couldn’t do the surgery and I had to wait 6-weeks because I was having eye surgery. So I had to wait. And so when I waited and then I went and he said, oh well it’s about time you got in here, we’ve been waiting a long time for this. And I went you’ve been waiting a long time? Like ah, how would you like to be at my end of the stick, I’m the one that’s got the problem here, not you. (SK)  I had infection up until maybe about a month ago. And he kept on saying, he looked at me from you and me, yah, you don’t have an infection. So I said, so the doctor, whatever…at the cancer clinic and he doesn’t know anything, he just know an infection when he sees one? I mean like it’s running out. I mean like you’d have to really blind not to see, all the stuff running out of there and all of the stuff they gave me to put on it and it was painful, it was, liked about this big I was so swollen, he was, that’s why he was downright ignorant actually. (SK) |
|  | Provided reassurance | She did a really good job of reassuring me that this isn’t some sort of crazy aggressive cancer that is going to spread through your body in two weeks. I wasn’t afraid that my surgery was a month away, I could wait and not have that fear. So that really helped me. (AB)  what I felt is that I have your back. Anything you need, I have your back. You know and if there are things that you don’t understand, come to me. (BC)  very good in that you know what, you can come back and see us if you can’t get into your family doctor; like we don’t want you waiting, we’ll make sure you get results. So he put me at ease in that it wasn’t gonna fall through the cracks. (AB)  She says were gonna get you through this, it’s gonna be okay. (SK) |
|  | Women reluctant to express emotions | My strategy is not seeking some emotional support from the medical system. I need to get this emotional support from somewhere else. (BC)  Only now, almost a year later I’m starting to actually find that it’s bothering me more than it did. At the time I just went into survival mode and it was like, I’m going to act like a normal person and pretend this isn’t happening. (AB)  I felt guilty because I kept thinking, I didn’t have chemo, I didn’t have radiation, what do I have to complain about? (ON)  I’ve had the paper that said, if you need emotional support come here but I also felt like a fraud contacting them. Now I’m treated, I should just move on, right? (AB) |
|  | Little supportive care | I felt that my doctor needed to leave but I still needed to process and the nurse navigator took me into a separate office and said, sit here for a few minutes, process, here’s some paper, why don’t you write down some questions and we’ll talk about it. (BC)  I guess if you have no knowledge she…like I found out from the mammogram place too because they called us and said has your physician contacted you yet? You need to come back in. So when I went in, I said, well obviously they need me to do something and she had no knowledge. So her reaction was very numb. She was very uncomfortable. I think this was her first diagnosis of any…of that sort. So she really didn’t…I was back in my car 5-minutes later calling my husband crying, right? Like…and that’s, no support from her… so that was frustrating (BC)  I don’t think I had a friendly, like there was no empathy between me and the medical professionals. This was more which one is gonna be better for me given my situation… So I felt really lucky that I had my science background because otherwise I could…I could not, I don’t understand how people navigate this (ON) |
| Managing uncertainty  *Identifying and providing information about uncertainty related to aspects of a medical condition, including side effects or life changes.* | Little information about prognosis | We never spoke about the likelihood of it turning into cancer…this was never a discussion. The way the conversation was framed was that it was cancer, it’s in the ducts and with time it will go out. (BC)  I was told that it was cancer. So really there was no uncertainty. (ON)  I’m not sure that he described any uncertainty with it, yah. I think this is what it is, like a very factual and this is what we need to do. (NS)  it wasn’t really a discussion because there wasn’t really a percentage I don’t think in her mind at that point (NS)  To be fair, I don’t remember about the details but from what I remember is that no, we never spoke about the not likelihood of it going into cancer. I…oh well, like this was never a discussion. I think the way the conversation was framed that its cancer, it’s in the ducts and with time it can go out. So it’s a matter of time. This is my understanding of the way the conversation was framed (BC)  Dr. X said, she never said it’s unlikely that this is…it was more like you’ve got multiple…this is in your left breast I…we’re gonna have to do…definitely take the left breast. Didn’t really feel like a lot of uncertainty because I think in a lot of people’s minds it was almost eminent that mine was gonna become invasive given the pathology and the history and everything; it was just…I was really lucky that it was caught when it was. (BC)  we talked about was well, how do you know it’s unlikely? So do you have any determinants? Are there any diagnostic? Are there any tumor markers? Like where do you as a mom of two kids, how do I say, I didn’t do any of this at all because I’m willing to take that risk and then two years from now end up with invasive ductal carcinoma. Why is it more unlikely for me than anyone else? And the answer…they really…they don’t know, they have no idea it’s a complete crap shoot. So you’re only option is deal with it unless you want to deal with it every year. (BC)  I do remember numbers were discussed. It was very clear, this is the uncertain stage of the diagnosis, it is known that a small fraction of people with DCIS will go onto become invasive. But it was clearly communicated in the context of statistics and the numbers of what we know, which was helpful when it came time to make a decision. (ON) |
| Making decisions  *Sharing and understanding preferences for involvement in decision-making; exploring options, discussing implications and assessing quality of choice in follow-up.* | Little involvement in discussion or decisions | You can’t change what your treatment plan is, you can only go with what they offer you. (NS)  They don’t say, hey are you a marathon runner? Are you this? Are you that? Do you go to the gym? Do you lift your kids? They don’t ask you any of that. (BC)  I was given both options but my doctor was really steering me towards partial mastectomy. (BC)  She wanted to make sure that I had good information about different options but also respected whichever decision I would make and guide me in that decision. (AB)  I don’t think I really had a choice because I was caught early enough, I wouldn’t need it…full blown mastectomy, it was just a partial, right? (BC)  because you are ignorant of everything…and read what they give you to read when your physician tells you that you have DCIS or whatever that you’re going to have this surgery, then you’re to go for radiation. You just follow what your treatment plan is. So when I had the DCIS and then I had the surgery then I was told I had to go over to the Cancer Treatment Centre and meet with the oncologist and they give you a package deal of how many radiation treatments you needed. So you just kind of go through the motions, right (NS)  So she laid out the treatment plan which was lumpectomy, radiation treatment. (NS)  She said I recommend that you have not a mastectomy, a lumpectomy. And I said, no, I want my breast off which my kids said, but if you don’t need it. But my thing was take it off and I won’t need anything else. But then she said, no and talked to me and said, there’s always time to do a mastectomy. Let’s see what when we send away, let’s see what happens. I would have preferred the mastectomy and not have to worry because… I feel if it’s not this one, next year it might be this one. But she was so calm and so reassuring and she’s the expert. So I said, okay I’m in your hands. If you tell me that I don’t need a mastectomy we’ll go your route. (ON)  I was sent back here because they told me that I didn’t need radiation, I needed a mastectomy. And I have to be honest when I went back to Dr. X and he said, you know I’m…he was almost crying actually. He said, I’m really sorry that…because I had asked him to take the breast and he said, I’m…I feel terrible…I just…he said, I really bad. And I could feel his emotion and I said, well it is what it is. I was a little disappointed I have to be quite honest with you; that they didn’t take the breast. (NS) |
|  | Uninformed and frustrated | I brought up mastectomy because I wanted to know. And she just simply said, it’s not appropriate for you which I was a little, honestly… but I wanted it to be appropriate for me, you know because still to this day, I don’t know, I’m not really that thrilled with radiation, right? To be honest. But I heard her and we proceeded. (NS)  it felt really uncomfortable in that I didn’t get the treatment I needed from them or the feeling that I needed from them…to understand what the diagnosis meant and what my options were. So it was a very quick meeting with them and I already had my surgery date booked for a mastectomy and I was like, wah, hold on, what? So that’s my experience. (BC)  When I did try to get more information from the office and from the people here, it was very disconnected and I never got phone calls back or I need to turn information. So I kind of felt like I had to go with what her recommendation was. In hind-sight, I’m glad but at the time I didn’t feel like I had enough knowledge and I had to just trust what she was telling me for face value. I have two kids as well, so I had to do something, couldn’t do nothing, that was one thing I did know. (BC)  I think there wasn’t enough information in that regard in terms of my choice in the diagnosis and in the procedure so. (BC)  they need to be…have better written information about the real implications of what you’re about to do whether it’s a mastectomy, whether it’s about the nipple, whether…I don’t…I don’t think they talk about that quite enough about you know what is radiation dermatitis how long…they’re still you may feel this, you may feel that, you may feel that, this would be here, you’ll…I think they could do a lot better at being much more factual upfront when they’re talking about what your options are. Because I think you don’t have the information and then you end up using your resource at where you are at any point in time in the process and not from the very beginning; so you have a better idea of what to expect. (BC)  he didn’t explain any advantages, disadvantages and like I said, he didn’t even understand when I said to him; well I’m really concerned about having radiation. He didn’t understand that. (SK) |
| Enabling self-management  *Helping to access and navigate resources relevant to needs; assisting with self-care strategies and following up to assess and advise.* | Little information about follow-up or self-care | I left here and I didn’t feel complete. I said, now what? (ON)  My experience was, you’re done treatment, bye-bye. That’s it. I was not happy about that. It would have been nice if she had said to me, you are now done, you’re going to feel such and such. (ON)  you’re not feeling well because you can’t lift your arms up. Like you know, you can’t do anything. Like if my mom hadn’t come out to help me with the drains and like I couldn’t move anything…I was not prepared for that at all. There was no discussion of like how impaired I’d be, zero. (BC)  There were some things I wished had been brought up which is…but after my mastectomy I wished someone would have told me about I’m going to the bathroom again, like for me that was a huge deal. I couldn’t eat, my feeling after the surgery was like I felt terrible for weeks and weeks. Like a lot of nausea and stuff that I didn’t really feel like I was really primed for. I don’t feel like I was given oodles of information about managing my after affects, just kind of like, laid out the bare bones of your gonna feel bad and don’t lift anything, etc., etc. I had really honestly two fairly small children and I think I was only able to lift whatever, really only lift like to waist level; that would have been atrocious for me and I think that’s something that could have been addressed. (AB)  So nobody knew who to take me. I was basically kind of forgotten. After some time I’m kind of like, so is that really it? (ON)  I don’t know what my follow-up is. I have no idea. What am I…was I supposed to go and have another …like what about this breast over here? Like I don’t know what my follow-up is. So that’s something I should find out but I’m not really sure, it’s not really my surgeron’s job. It’s not really the plastic surgeon’s job. So…and BC Cancer I’ve only ever met with them once. So I don’t really know where I’m supposed to go… I guess. (BC)  Actually I haven’t seen Dr. X after the…on the day of the surgery didn’t see her and then it was only two and half months after that they called for a meeting. So I wasn’t sure what came out of the result of the surgery, after…until two and half months later to see if there was something. So follow-up and stuff I think…it’s…a messed up process, so many people are involved, so I didn’t have one contact person. (BC)  I just finished radiation last week, so I don’t know what the plan is going forward. (BC)  because I had the immediate reconstruction I was a little bit confused to as to who was in charge of what. Like do I follow-up with my surgeon? Do I go to my plastic surgeon? Like who do I ask or I’ve never had a clear sense of do I go to my family doctor for this or do I go back to my surgeon or do I phone the patient navigator or. So I was quite confused about who to go to when I needed what kind of help. (AB)  You know and when I look back, I think that was probably the worst part of the experience was the complete lack of support. And to be honest with you I’ve had no follow-up with my health centre other than you know other than from the big cancer agency. So no support (BC)  Definitely no follow-up and no kind of here’s the…here call us if you, you know, they were great when I was in the office….like I had to go home and decompress but it would have been nice to have somebody I could call afterwards. And there are a lot of agencies in town, there are a lot of places but that wasn’t at anybody’s fingertips. Just like afterwards there are a lot of physiotherapists and trainers now who are specializing in getting people back on their feet after and none of that was available when asked so. (BC)  in terms of support like there isn’t really any support for us with DCIS that I can find because precisely because of this pre-cancer language. Like people are like oh, well you didn’t really have cancer or you don’t…and it’s like, no you don’t understand. Like this…because when you have a family history of it and like then I was diagnosed it’s traumatic, it really is. It’s really hard and I don’t feel like there’s a whole lot of support out there. (BC)  But I mean if you look you can find stuff but it would be nice to get this little card going this is your diagnosis, these are the things you might want to tap into; they’re there for you (SK)  I actually had to phone around to find out who was still looking after me. So yah, I phoned the surgeon’s offices, the oncologists, like everywhere. And one actually said, well you missed your mammogram last year. And I went no I didn’t. I had it and she looked it up, oh yah, it was still left open. (ON)  I found out about breast cancer supportive care from a friend of mine that had been referred there by her family doctor. So I did, I seeked that out on my own and that’s where I probably got the most help with. (AB) |
|  | Patient navigators helped to reduced worry | What I found really useful here was they gave us a number for the nurse navigator…so anytime you had a problem or anything like that, you could phone her which was excellent. I really appreciated having her because sometimes you don’t know, you can’t phone the doctor. So if you have a person that can say, oh yah, come in we can squeeze you here, that’s very helpful (SK)  I love nurse navigators. And she was easy to call, easy to ask questions, sort of ran into inference for things and she got me a referral to the physio because I wanted it personally. (SK)  I think I was still a little emotional. She gave me the contact information for a nurse navigator. She automatically gave me that and she was like…please don’t try to do this all by yourself. Because I… she had encouraged me to take time off and I declined it. (ON)  come in and talk if you need too or like the nurse here at the breast clinic was amazing and she said…Anytime call me, email me, if you need to see me just come in. Like I’m always available…and she was, like when I was like dealing with drains and stuff like that, like I’d have a question, I’d pick up the phone and she was right there. so I knew the support was there if I was gonna need it. (NS)  I think having the nurse, access to the nurse navigator helped too. she spent a lot of time on the phone talking, she probably spent half an hour which was awesome. But then she’s like you know what, let’s make an appointment to follow-up, she’s like because were gonna hang up the phone and you’re gonna think of a whole bunch of things to ask. So then it was reassuring in that when I hung up the phone, already knew where my next step was. So that was a help too I think. (AB)  she could see I was still…so she…I think she even explained the next steps of treatment and I was still upset and she could see that. And so she asked me if it would be helpful to me if she had a nurse come in and talk about the diagnosis. And I said, yes. And so we did some…so the nurse came in and we had a conversation. So that was really helpful. (NS) |
